# Supplementary material for: Association of Two Indices of Insulin Resistance Marker with Abnormal Liver Function Tests: A Cross-Sectional Population Study in Taiwanese Adults
Source: Medicina (Kaunas). 2021 Dec 21;58(1):4. doi: 10.3390/medicina58010004 (PMC8781419; doi:10.3390/medicina58010004)
Supplement: Supplementary file 1 [file medicina-58-00004-s001.zip › Supplementary Figure S2.pdf]

Association of two indexes of insulin resistance marker with abnormal liver function biomarkers: a cross-sectional population study in Taiwanese adults

Adi Lukas Kurniawan[1,*], Chien-Yeh Hsu [2,3], Jane C.-J. Chao [3,4,5,*], Rathi Paramastri [4], Hsiu-An Lee [6,7], and Amadou-Wurry Jallow [8]

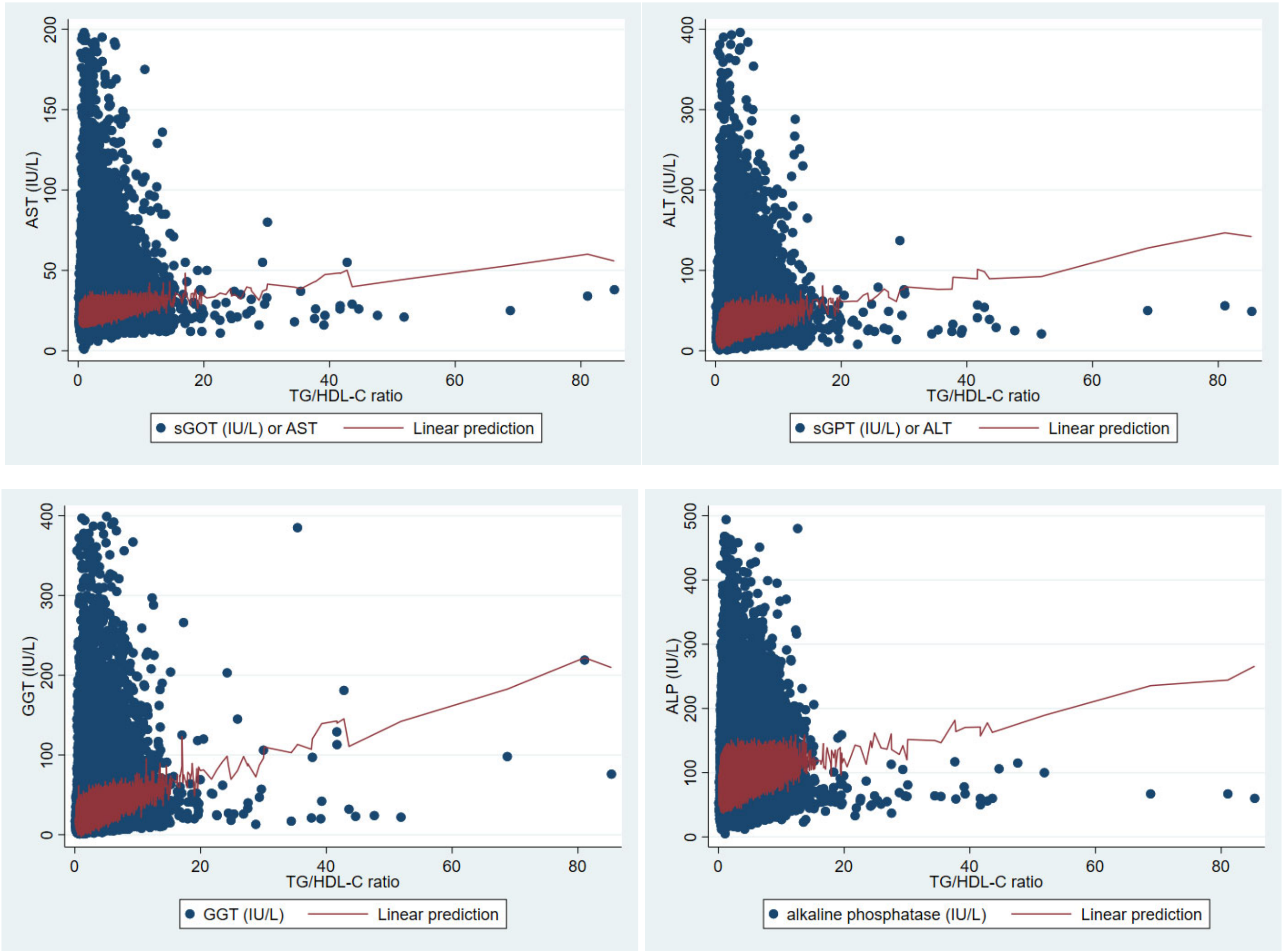

Figure S2. Linear prediction of TG/HDL-C ratio with serum liver function.
