# Supplementary material for: Association of Two Indices of Insulin Resistance Marker with Abnormal Liver Function Tests: A Cross-Sectional Population Study in Taiwanese Adults
Source: Medicina (Kaunas). 2021 Dec 21;58(1):4. doi: 10.3390/medicina58010004 (PMC8781419; doi:10.3390/medicina58010004)
Supplement: Supplementary file 1 [file medicina-58-00004-s001.zip › Supplementary Table S1.pdf]

Association of two indexes of insulin resistance marker with abnormal liver function biomarkers: a cross-sectional population study in Taiwanese adults

Adi Lukas Kurniawan[1,*], Chien-Yeh Hsu [2,3], Jane C.-J. Chao [3,4,5,*], Rathi Paramastri [4], Hsiu-An Lee [6,7], and Amadou-Wurry Jallow [8]

Table S1. Factor loading of three dietary patterns identified with principal component analysis

Food groups	Dietary pattern		
	**Western style**	**Vege-seafood style**	**American breakfast style**
milk			0.602
dairy products			0.671
eggs	0.443		
meat	0.570		
seafood		0.375	
innards organs	0.463		
beans/bean products		0.354	
light colored vegetables		0.805	
dark colored vegetables		0.824	
fried vegetables/salad dressing		0.614	
fruits		0.433	0.335
rice/flour products		0.303	
whole grains			0.404
fried rice/flour products	0.463		
root crops		0.366	0.387
breads	0.312		0.485
jam/honey			0.353
sugar	0.514		
deep fried foods	0.688		
preserved vegetables, processed meat or fish	0.589		
instant noodles	0.414		
soy sauce	0.572		

Factor loading below 0.30 was not shown in the table for simplicity.
