# Supplementary material for: Association of Two Indices of Insulin Resistance Marker with Abnormal Liver Function Tests: A Cross-Sectional Population Study in Taiwanese Adults
Source: Medicina (Kaunas). 2021 Dec 21;58(1):4. doi: 10.3390/medicina58010004 (PMC8781419; doi:10.3390/medicina58010004)
Supplement: Supplementary file 1 [file medicina-58-00004-s001.zip › Supplementary Table S2.pdf]

Association of two indexes of insulin resistance marker with abnormal liver function biomarkers: a cross-sectional population study in Taiwanese adults

Adi Lukas Kurniawan[1,*], Chien-Yeh Hsu [2,3], Jane C.-J. Chao [3,4,5,*], Rathi Paramastri [4], Hsiu-An Lee [6,7], and Amadou-Wurry Jallow [8]

Table S2. Multivariable linear regression of liver function tests according to individual fasting blood glucose, triglyceride, and HDL-C levels

	**AST (IU/L)**		**ALT (IU/L)**		**GGT (IU/L)**		**ALP (IU/L)**	
	**β (95% CI)**	**AUC (95% CI)**	**β (95% CI)**	**AUC (95% CI)**	**β (95% CI)**	**AUC (95% CI)**	**β (95% CI)**	**AUC (95% CI)**
FBG (mmol/L)	0.11 (0.04, 0.18)**	0.636 (0.629 – 0.643)**	0.81 (0.68, 0.95)**	0.643 (0.639 – 0.648)**	1.33 (1.18, 1.48)**	0.655 (0.651 – 0.659)**	2.35 (2.02, 2.68)**	0.537 (0.533 – 0.540)**
TG (mmol/L)	0.60 (0.51, 0.70)**	0.686 (0.680 – 0.693)**	2.76 (2.58, 2.94)**	0.729 (0.725 – 0.733)**	5.01 (4.80, 5.21)**	0.742 (0.739 – 0.745)**	4.83 (4.39, 5.28)**	0.586 (0.582 – 0.590)**
HDL-C (mmol/L)	-0.74 (-0.95, -0.52)**	0.604 (0.597 – 0.611)**	-4.93 (-5.35, -4.51)	0.677 (0.673 – 0.681)**	-9.49 (-9.96, -9.02)**	0.636 (0.632 – 0.640)**	-17.84 (-18.85, -16.82)**	0.564 (0.560 – 0.568)**

Data are adjusted by age and gender, BMI, body fat, WHR, marital status, education level, physical activity status, income status, smoking, alcohol drinking, sleeping status (condition and time), hypertension, diabetes, cardiovascular disease status, hyperuricemia, reduced kidney function, high inflammation, T-Cholesterol, LDL-C levels, and all type of dietary pattern scores (model 2).

** $p < 0.01$
