# Supplementary material for: Association of Two Indices of Insulin Resistance Marker with Abnormal Liver Function Tests: A Cross-Sectional Population Study in Taiwanese Adults
Source: Medicina (Kaunas). 2021 Dec 21;58(1):4. doi: 10.3390/medicina58010004 (PMC8781419; doi:10.3390/medicina58010004)
Supplement: Supplementary file 1 [file medicina-58-00004-s001.zip › Supplementary Table S3.pdf]

Association of two indexes of insulin resistance marker with abnormal liver function biomarkers: a cross-sectional population study in Taiwanese adults

Adi Lukas Kurniawan[1,*], Chien-Yeh Hsu [2,3], Jane C.-J. Chao [3,4,5,*], Rathi Paramastri [4], Hsiu-An Lee [6,7], and Amadou-Wurry Jallow [8]

Table S3. Multivariable adjusted linear regression for liver function tests based on TyG index and TG/HDL-C ratio

	AST (IU/L)		ALT (IU/L)		GGT (IU/L)		ALP (IU/L)	
	**Model 1**	**Model 2**	**Model 1**	**Model 2**	**Model 1**	**Model 2**	**Model 1**	**Model 2**
TyG index	1.53 (1.41 – 1.65)**	0.68 (0.55 – 0.81)**	5.09 (4.86 – 5.32)**	3.70 (3.45 – 3.95)**	8.58 (8.32 – 8.84)**	6.05 (5.77 – 6.33)**	10.66 (10.10 – 11.22)**	10.16 (9.56 – 10.76)**
TG/HDL-C ratio	0.43 (0.40 – 0.47)**	0.23 (0.20 – 0.27)**	1.39 (1.32 – 1.46)**	1.08 (1.01 – 1.15)**	2.18 (2.10 – 2.26)**	1.62 (1.54 – 1.70)**	2.62 (2.45 – 2.79)**	2.18 (2.01 – 2.36)**

Data are expressed as beta (β) and 95% confidence intervals (CIs) in the parenthesis

Model 1: adjusted by age and gender, BMI, body fat, WHR, marital status, education level, physical activity status, income status, smoking, alcohol drinking, sleeping status (condition and time), hypertension, diabetes, and cardiovascular disease status.

Model 2: adjusted by model 1 + hyperuricemia, reduced kidney function, high inflammation, T-Cholesterol, LDL-C levels, and all type of dietary pattern scores.

** $p < 0.01$
