# Supplementary material for: Association of Two Indices of Insulin Resistance Marker with Abnormal Liver Function Tests: A Cross-Sectional Population Study in Taiwanese Adults
Source: Medicina (Kaunas). 2021 Dec 21;58(1):4. doi: 10.3390/medicina58010004 (PMC8781419; doi:10.3390/medicina58010004)
Supplement: Supplementary file 1 [file medicina-58-00004-s001.zip › Supplementary Table S4.pdf]

Association of two indexes of insulin resistance marker with abnormal liver function biomarkers: a cross-sectional population study in Taiwanese adults

Adi Lukas Kurniawan[1,*], Chien-Yeh Hsu [2,3], Jane C.-J. Chao [3,4,5,*], Rathi Paramastri [4], Hsiu-An Lee [6,7], and Amadou-Wurry Jallow [8]

Table S4. Multivariable adjusted logistic regression for abnormal liver function in different subgroups according to quintiles of TyG index and TG/HDL-C ratio

	Q3	High AST			High ALT			High GGT			High ALP		
		Q1	Q2	Q4	Q2	Q3	Q4	Q2	Q3	Q4	Q2	Q3	Q4
**TyG index**													
Inflammation													
Normal	1.00	0.85 (0.75 – 0.97)*	0.87 (0.78 – 0.98)*	1.07 (0.97 – 1.18)	0.70 (0.63 – 0.78)**	0.80 (0.74 – 0.87)**	1.28 (1.20 – 1.37)**	0.60 (0.55 – 0.65)**	0.80 (0.74 – 0.85)**	1.38 (1.30 – 1.46)**	0.55 (0.52 – 0.58)**	0.83 (0.79 – 0.87)**	1.07 (1.02 – 1.12)**
High	1.00	0.95 (0.73 – 1.23)	0.86 (0.69 – 1.08)	1.06 (0.88 – 1.26)	0.67 (0.54 – 0.83)**	0.75 (0.63 – 0.90)**	1.11 (0.97 – 1.28)	0.63 (0.53 – 0.76)**	0.78 (0.68 – 0.91) **	1.28 (1.14 – 1.43)**	0.59 (0.52 – 0.68)**	0.87 (0.77 – 0.97)*	1.04 (0.94 – 1.16)
Uric acid													
Normal	1.00	0.91 (0.80 – 1.04)	0.92 (0.82 – 1.03)	1.09 (0.98 – 1.21)	0.72 (0.65 – 0.80)**	0.83 (0.76 – 0.91)**	1.25 (1.15 – 1.34)**	0.61 (0.56 – 0.66)**	0.80 (0.75 – 0.86)**	1.35 (1.27 – 1.44)**	0.57 (0.54 – 0.61)**	0.85 (0.81 – 0.90)**	1.08 (1.03 – 1.14)**
Hyperuricemia	1.00	0.73 (0.56 – 0.95)*	0.76 (0.62 – 0.92)**	1.03 (0.90 – 1.19)	0.61 (0.49 – 0.75)**	0.68 (0.59 – 0.79)**	1.25 (1.13 – 1.38)**	0.59 (0.49 – 0.71)**	0.75 (0.66 – 0.86)**	1.36 (1.24 – 1.49)**	0.59 (0.52 – 0.67)**	0.82 (0.74 – 0.90)**	1.01 (0.93 – 1.09)
BMI													
Normal	1.00	0.88 (0.77 – 1.01)	0.88 (0.77 – 1.00)	1.07 (0.94 – 1.22)	0.74 (0.66 – 0.83)**	0.81 (0.73 – 0.91)**	1.26 (1.14 – 1.38)**	0.63 (0.58 – 0.69)**	0.83 (0.77 – 0.90)**	1.39 (1.29 – 1.49)**	0.56 (0.53 – 0.59)**	0.82 (0.78 – 0.87)**	1.07 (1.01 – 1.14)*
Over/obese	1.00	0.75 (0.59 – 0.94)*	0.85 (0.73 – 1.00)	1.07 (0.96 – 1.20)	0.63 (0.53 – 0.74)**	0.77 (0.69 – 0.87)**	1.22 (1.12 – 1.32)**	0.53 (0.45 – 0.62)**	0.73 (0.66 – 0.81)**	1.29 (1.20 – 1.39)**	0.65 (0.57 – 0.74)**	0.91 (0.83 – 1.00)	1.07 (0.99 – 1.15)
Age group													
30 – 45 y	1.00	0.88 (0.76 – 1.02)	0.89 (0.78 – 1.02)	1.10 (0.98 – 1.23)	0.76 (0.68 – 0.84)**	0.82 (0.75 – 0.90)**	1.28 (1.19 – 1.38)**	0.68 (0.62 – 0.74)**	0.84 (0.78 – 0.91)**	1.43 (1.34 – 1.53)**	0.56 (0.52 – 0.59)**	0.83 (0.79 – 0.88)**	1.06 (1.00 – 1.12)*
> 45 y	1.00	1.08 (0.91 – 1.29)	0.91 (0.78 – 1.06)	1.01 (0.89 – 1.14)	0.85 (0.72 – 1.02)	0.82 (0.72 – 0.95)**	1.11 (1.00 – 1.24)*	0.59 (0.51 – 0.69)**	0.76 (0.68 – 0.85)**	1.21 (1.11 – 1.31)**	0.59 (0.54 – 0.66)**	0.85 (0.79 – 0.93)**	1.08 (1.01 – 1.16)*
Gender													
Men	1.00	1.03 (0.84 – 1.27)	0.85 (0.72 – 1.00)	1.00 (0.88 – 1.13)	0.69 (0.60 – 0.81)**	0.81 (0.72 – 0.90)**	1.24 (1.14 – 1.34)**	0.62 (0.54 – 0.71)**	0.84 (0.76 – 0.93)**	1.42 (1.32 – 1.53)**	0.57 (0.51 – 0.64)**	0.82 (0.75 – 0.89)**	1.11 (1.03 – 1.19)**
Women	1.00	0.81 (0.71 –	0.88 (0.78 –	1.11 (0.99 –	0.67 (0.60 –	0.77 (0.69 –	1.24 (1.12 –	0.56 (0.51 –	0.75 (0.69 –	1.26 (1.17 –	0.57 (0.54 –	0.85 (0.800	1.05 (0.99 –

		0.93)**	1.00)	1.25)	0.76)**	0.86)**	1.36)**	0.61)**	0.81)**	1.36)**	0.61)**	– 0.90)**	1.11)
**TG/HDL-C ratio**													
Inflammation													
Normal	1.00	0.92 (0.81 – 1.04)	0.92 (0.82 – 1.03)	1.14 (1.03 – 1.25)**	0.78 (0.71 – 0.87)**	0.80 (0.73 – 0.87)**	1.30 (1.22 – 1.40)**	0.60 (0.55 – 0.65)**	0.78 (0.73 – 0.84)**	1.35 (1.27 – 1.43)**	0.61 (0.57 – 0.64)**	0.86 (0.82 – 0.91)**	1.11 (1.05 – 1.17)**
High	1.00	0.92 (0.72 – 1.17)	0.83 (0.67 – 1.03)	0.99 (0.84 – 1.17)	0.82 (0.66 – 1.01)	0.86 (0.72 – 1.02)	1.06 (0.93 – 1.21)	0.67 (0.56 – 0.80)**	0.82 (0.71 – 0.94)**	1.20 (1.07 – 1.35)**	0.61 (0.54 – 0.70)**	0.92 (0.82 – 1.04)	1.10 (0.99 – 1.22)
Uric acid													
Normal	1.00	0.91 (0.80 – 1.03)	0.89 (0.79 – 1.00)	1.09 (0.98 – 1.21)	0.80 (0.73 – 0.89)**	0.84 (0.76 – 0.92)**	1.24 (1.15 – 1.34)**	0.62 (0.57 _ 0.67)**	0.79 (0.74 – 0.85)**	1.30 (1.22 – 1.39)**	0.61 (0.58 – 0.65)**	0.87 (0.83 – 0.92)**	1.12 (1.06 – 1.18)**
Hyperuricemia	1.00	0.86 (0.66 – 1.11)	0.91 (0.75 – 1.10)	1.12 (0.98 – 1.29)	0.68 (0.55 – 0.85)**	0.71 (0.61 – 0.83)**	1.27 (1.14 – 1.41)**	0.57 (0.48 – 0.69)**	0.79 (0.69 – 0.90)**	1.35 (1.23 – 1.48)**	0.64 (0.56 – 0.72)**	0.90 (0.82 – 1.00)	1.06 (0.98 – 1.15)
BMI													
Normal	1.00	0.81 (0.71 – 0.93)**	0.82 (0.72 – 0.93)**	1.07 (0.94 – 1.22)	0.79 (0.70 – 0.88)**	0.77 (0.69 – 0.86)**	1.25 (1.13 – 1.37)**	0.61 (0.56 – 0.67)**	0.81 (0.75 – 0.88)**	1.30 (1.21 – 1.40)**	0.61 (0.57 – 0.64)**	0.84 (0.80 – 0.89)**	1.10 (1.04 – 1.16)**
Over/obese	1.00	0.87 (0.70 – 1.08)	0.98 (0.84 – 1.14)	1.13 (1.01 – 1.26)*	0.71 (0.60 – 0.84)**	0.87 (0.77 – 0.98)*	1.24 (1.14 – 1.34)**	0.58 (0.49 – 0.67)**	0.75 (0.68 – 0.83)**	1.31 (1.22 – 1.41)**	0.70 (0.62 – 0.79)**	1.01 (0.92 – 1.11)	1.14 (1.06 – 1.22)**
Age group													
30 – 45 y	1.00	0.86 (0.74 – 1.00)	0.89 (0.78 – 1.03)	1.09 (0.97 – 1.22)	0.79 (0.71 – 0.89)**	0.77 (0.69 – 0..85)**	1.26 (1.16 – 1.36)**	0.64 (0.59 – 0.71)**	0.77 (0.71 – 0.84)**	1.33 (1.25 – 1.43)**	0.59 (0.56 – 0.63)**	0.82 (0.78 – 0.87)**	1.09 (1.03 – 1.16)**
> 45 y	1.00	1.06 (0.91 – 1.24)	0.93 (0.80 – 1.07)	1.10 (0.98 – 1.24)	0.93 (0.80 – 1.08)	0.93 (0.82 – 1.05)	1.19 (1.08 – 1.32)**	0.62 (0.55 – 0.70)**	0.85 (0.77 – 0.93)**	1.27 (1.17 – 1.38)**	0.66 (0.60 – 0.72)**	0.97 (0.90 – 1.05)	1.13 (1.05 – 1.21)**
Gender													
Men	1.00	1.13 (0.91 – 1.41)	0.91 (0.77 – 1.09)	0.99 (0.87 – 1.13)	0.77 (0.65 – 0.92)**	0.76 (0.68 – 0.86)**	1.22 (1.12 – 1.33)**	0.62 (0.53 – 0.72)**	0.79 (0.71 – 0.87)**	1.37 (1.27 – 1.48)**	0.63 (0.56 – 0.71)**	0.85 (0.78 – 0.93)**	1.15 (1.07 – 1.240**
Women	1.00	0.85 (0.75 – 0.97)*	0.88 (0.78 – 0.99)*	1.18 (1.06 – 1.32)**	0.75 (0.67 – 0.84)**	0.82 (0.74 – 0.91)**	1.26 (1.15 – 1.38)**	0.58 (0.53 – 0.63)**	0.77 (0.71 – 0.83)**	1.26 (1.17 – 1.35)**	0.61 (0.57 – 0.65)**	0.88 (0.83 – 0.93)**	1.07 (1.01 – 1.14)**

Data are expressed as odd ratio (OR) and 95% confidence intervals (CIs) in the parenthesis
Adjusted by Model 2: age and gender, BMI, body fat, WHR, marital status, education level, physical activity status, income status, smoking, alcohol drinking, sleeping status (condition and time), hypertension, diabetes, cardiovascular disease status, hyperuricemia, reduced kidney function, high inflammation, T-Cholesterol, and LDL-C levels, and all type of dietary pattern scores.
* $p < 0.05$, ** $p < 0.001$
